# Rhizosphere enzyme activities and microorganisms drive the transformation of organic and inorganic carbon in saline–alkali soil region

**DOI:** 10.1038/s41598-022-05218-7

**Published:** 2022-01-25

**Authors:** Yunke Qu, Jie Tang, Ben Liu, Hang Lyu, Yucong Duan, Yao Yang, Sining Wang, Zhaoyang Li

**Affiliations:** 1 Key Lab of Groundwater Resources and Environment, Ministry of Education, Jilin University, Changchun, 130012 China; 2 College of New Energy and Environment, Jilin University, Changchun, 130012 China

**Keywords:** Agroecology, Boreal ecology, Ecology, Environmental sciences

## Abstract

Western Jilin Province is one of the world's three major saline–alkali land distribution areas, and is also an important area of global climate change and carbon cycle research. Rhizosphere soil microorganisms and enzymes are the most active components in soil, which are closely related to soil carbon cycle and can reflect soil organic carbon (SOC) dynamics sensitively. Soil inorganic carbon (SIC) is the main existing form of soil carbon pool in arid saline–alkali land, and its quantity distribution affects the pattern of soil carbon accumulation and storage. Previous studies mostly focus on SOC, and pay little attention to SIC. Illumina Miseq high-throughput sequencing technology was used to reveal the changes of community structure in three maize fields (M1, M2 and M3) and three rice fields (R1, R2 and R3), which were affected by different levels of salinization during soil development. It is a new research topic of soil carbon cycle in saline–alkali soil region to investigate the effects of soil microorganisms and soil enzymes on the transformation of SOC and SIC in the rhizosphere. The results showed that the root—soil—microorganism interaction was changed by saline–alkali stress. The activities of catalase, invertase, amylase and *β*-glucosidase decreased with increasing salinity. At the phylum level, most bacterial abundance decreases with increasing salinity. However, the relative abundance of *Proteobacteria* and *Firmicutes* in maize field and *Firmicutes*, *Proteobacteria* and *Nitrospirae* in rice field increased sharply under saline–alkali stress. The results of redundancy analysis showed that the differences of rhizosphere soil between the three maize and three rice fields were mainly affected by ESP, pH and soil salt content. In saline–alkali soil region, *β*-glucosidase activity and amylase were significantly positively correlated with SOC content in maize fields, while catalase and *β*-glucosidase were significantly positively correlated with SOC content in rice fields. *Actinobacteria*, *Bacteroidetes* and *Verrucomicrobia* had significant positive effects on SOC content of maize and rice fields. *Proteobacteria*, *Gemmatimonadetes* and *Nitrospirae* were positively correlated with SIC content. These enzymes and microorganisms are beneficial to soil carbon sequestration in saline–alkali soils.

## Introduction

Soil carbon pool is the largest carbon pool in terrestrial ecosystems. Its dynamic change and driving mechanism are the focus and hotspot of terrestrial ecosystem carbon cycle and global change research, and also one of the core issues of global change research programs such as the Global Carbon Project (GCP) and World Climate Research Program (WCRP)^1^. Soil carbon pool mainly includes soil organic carbon (SOC) pool and inorganic carbon (SIC) pool. SOC plays a dominant role in soil carbon pool in humid areas, while SIC is the main form of carbon in arid and semi-arid areas with annual rainfall less than 400mm^2,3^. It is estimated that the global SOC pool is as high as 1.4–1.5 × 10^18^ g C, which is about 2.4 times of the total terrestrial biological carbon and 3 times of the total atmospheric carbon^4^. A change of 0.1% in soil carbon pool will increase atmospheric CO_2_ concentration by 1 mg m^−3^ and have a profound impact on global climate change.

Soil enzymes are ubiquitous in soil and can be considered as one of the most active components in soil, which can promote the mineralization and decomposition of SOM and release inorganic nutrients^5–7^. Soil pH directly affects the speed at which soil enzymes participate in biochemical reactions, and when pH exceeds its optimum range, enzyme activity will be inhibited^8^. Studies have shown that oxidase activity is more affected by soil pH^9^. Zhang et al.^10^ found that soil enzyme activity was also affected by saline–alkali stress, and soil salt content (SSC) in salinized soil was significantly negatively correlated with enzyme activities such as hydrooxidase and invertase. The relationship between soil pH and enzyme activity also has a controlling effect on SOC^11^. From a global perspective, Sinsabaugh et al.^12^. concluded that hydrolase activity was more related to SOC. This finding suggests that hydrolases may be more important for the decomposition of SOC, with implications for nutrients and carbon cycling. Marx et al.^13^ studied the distribution of hydrolases related to soil nutrients and found that the enzyme activity related to carbon cycling was the highest in coarse sand. The study on the activity changes of various enzymes is of great significance for revealing the process of SOM transformation.

Soil microorganism plays a key role in regulating matter circulation of ecosystem and is an important index to measure soil properties and functions^14^. The exchange of matter and energy between soil, rhizosphere micr oorganisms, and plants forms a close and special relationship, which makes the abundance and species of rhizosphere microorganisms differ to some extent from those of non-rhizosphere soils^15^. Soil physico-chemical properties, nutrient cycling and microbial activity are affected by the type of soil tillage^16^. The changes of soil temperature, moisture and carbon input will also have a great effect on soil microbial activity, which in turn will affect the nutrient availability due to the turnover of SOM^17^. Lenton et al.^18^ showed that the increase of temperature led to the change of microbial community structure, which accelerated the decomposition rate of SOC and released the carbon stored in the soil into the atmosphere. Rey et al.^19^ found that moisture changes the oxygen condition and microbial activity of soil environment, thus affecting SOC mineralization.

In recent years, the northeast of China has been increasingly affected by global climate change and human disturbance, which has exacerbated the process of soil salinization and desertification in Songnen Plain^20^. Land salinization has become one of the world's resource and environmental problems, which is a huge environmental pressure restricting the development of agriculture and causes billions of agricultural economic losses every year. Soil salinization is a kind of land degradation caused by excessive accumulation of soil salinization under the combined action of natural and human factors^21^. It can damage the normal growth of plants, change the structure and function of cell membrane, and have toxic effects on cells. Western Jilin Province, located in the Northeast China, is a typical vulnerable area for global carbon cycle research, and also one of the three major concentrated distribution areas of soda saline–alkali soil in the world^22,23^. Previous studies on soil carbon pool mainly focused on forest, wetland and grassland soils. However, the research on soil carbon pool in saline–alkali land is relatively weak, especially the SIC and the effects of soil enzymes and microorganisms is not clear^24–26^.

After decades of cultivation and development, a special saline alkali agroecosystem has been formed in the study area, with maize and rice as the main crops. We selected the rhizosphere soil of maize and rice as the research object. The aim of this study is to analyze the variation of physico-chemical properties, soil enzyme activities and microbial community structure of maize and rice rhizosphere soil and their effects of soil enzymes and microorganisms on the transformation of SOC and SIC in soda saline–alkali fields. It provides a basic example for the study of global carbon cycle in saline–alkali farmland, which is of great significance to the protection of saline–alkali land resources and the sustainable development of agriculture.

## Materials and methods

### Study area description

**Figure 1:**
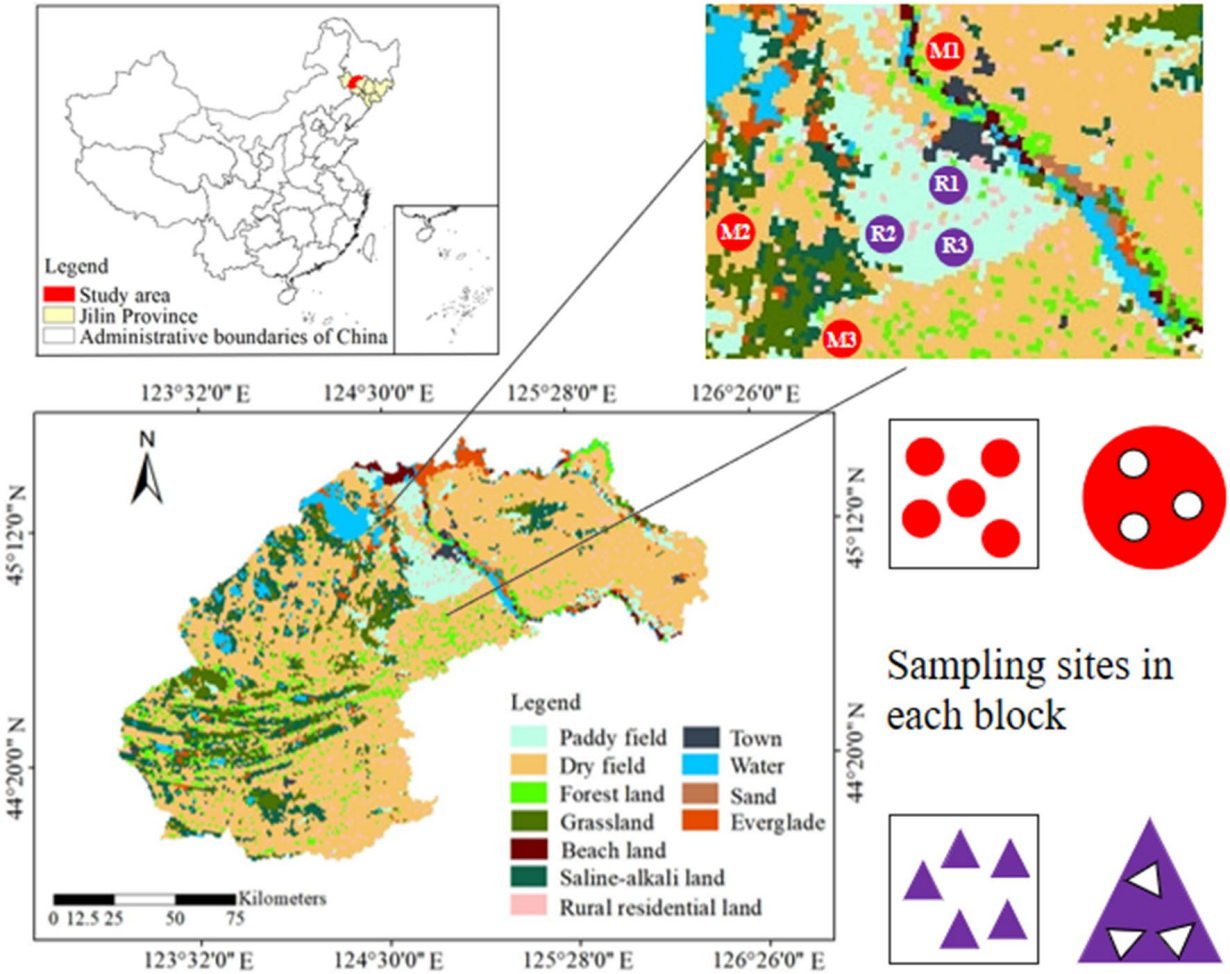
Location map of sampling sites in the study area (Songyuan).

The study area is located in Songyuan City, which belongs to the west of Jilin Province (Fig. 1). The area has experienced multiple desertification and saline–alkali desertification evolution process, forming a large area of saline–alkali soil deposition^27^. The region is a semi-arid and semi humid continental monsoon climate zone, with obvious difference in four seasons. The average annual precipitation is 558.3 mm, and the highest average temperature in July is 23.5 °C.

### Soil sampling and experimental design

Maize and rice are the main crops in the study area, accounting for 90.12% of the total grain output in Songyuan City. They are easy to be planted on a large scale and have relatively high economic benefits. Therefore, we selected the rhizosphere soil of these two crops for study. In order to make the sample plots more representative and the test results more universal, we took samples according to the soil type map and land use type map, combined with field investigation. Maize and rice are both monocropped, sown in May and harvested in October. The fertilization and management measures of all the fields were consistent, and the fertilizers (urea, potassium and phosphorus) were applied once on May 8.

Before sowing in April, we collected a large number of soil samples around Songyuan City and took them back to the laboratory for physico-chemical properties testing. We selected three maize fields and three rice fields with different levels of salinity during soil development, and the soil type was salinized meadow soil (Table 1). The second field sampling of rhizosphere soil was collected on August 16, 2019 (vigorous root growth stage), and 3 parallel sampling points were randomly selected at each site. We set up five sampling blocks (15 m × 15 m) in each sampling field, and each sampling block contains three parallel sampling sites (Fig. 1). When sampling, all the roots of plants were dug out. Due to the difference between paddy fields and maize fields, we assume that the soil attached to root system is regarded as rhizosphere soil. Subsamples from the same block were mixed to generate a composite sample. A total of 30 composite samples of maize and rice were placed in sterile sealed bags, stored in incubators filled with ice, and quickly transported to the laboratory. The composite samples were divided into three parts: one part was ground after natural air drying in the laboratory, screened by 2 mm and 0.2 mm respectively, and bagged for standby; some parts were refrigerated at 4 °C for microbial enzyme activity test; the other part was stored at − 80 °C for microbial community structure measurement.

**Table 1:** Background information on sampling sites.

Samplingsites	Soil classifification	pH	Clay (%)	Silt (%)	Sand (%)	Fertilization treatment	Experimentalarea	Type of crop
M1	Loam	8.43	14.03	40.73	45.24	N, P and K	95 m × 100 m	Maize
M2	Silty loam	9.23	8.52	73.48	18.00	N, P and K	99 m × 103 m	Maize
M3	Sandy loam	9.98	7.94	24.98	47.08	N, P and K	90 m × 100 m	Maize
R1	Loam	8.23	14.07	40.18	45.75	N, P and K	98 m × 102 m	Rice
R2	Silty loam	9.37	10.75	67.33	21.92	N, P and K	97 m × 101 m	Rice
R3	Sandy loam	9.94	4.58	31.30	64.12	N, P and K	95 m × 99 m	Rice

### Physico-chemical analysis of soil

Soil pH, soil salt content(SSC), Alkali-N (AN), were determined on soil passed through the 2 mm sieve. SOM was determined on soil passed through the 0.2 mm sieve. Soil physico-chemical properties were determined following the methods described by Zheng^28^. Soil texture was measured by Mastersizer 2000 laser particle size analyzer. Soil pH and SSC were determined by pH meter and residue drying—mass method in a ratio of 5–1 (water to soil), respectivily. SOM was determined by oil bath heating dichromate oxidation method. The concentration of exchangeable sodium (c mol (Na^+^) kg^−1^) was measured by using flame photometry (Shimadzu optical doublebeam atomic absorption spectrophotometer, Shanghai). Cation exchange capacity (CEC) (c mol kg^−1^) was determined by the EDTA-ammonium acetate salt exchange method. The exchangeable sodium percentage (ESP) was calculated by the following formula.

$${\text{ESP = Na }}^{ + } {\text{/CEC}}\; \times \;{100}$$

### Soil enzyme activities

Catalase activity (EC 1.11.1.6) was determined by potassium permanganate titration and was calculated after the blank subtraction according to the volume of consuming of KMnO_4_ standard solution. Soil invertase (EC 3.2.1.26) and amylase (EC 3.2.1.2) activities were determined by 3,5-Dinitrosalicylic acid colorimetry. Both of them were analyzed by spectrophotometry at 508 nm, and the activity of invertase and amylase could be expressed as mg g^−1^ according to the amount of reducing sugar generated after one day of culture. The soil *β*-glucosidase activity (EC3.2.1.21) was measured by using p-nitrophenol-*β*-D-glucoside as substrate, p-nitrophenol was hydrolyzed to produce p-nitrophenol under the action of *β*-glucosidase, and the activity of *β*-glucosidase was colorimetrically determined at 400 nm.

### Soil microbial community analysis

Soil genomic DNA was extracted from the sample using the E.Z.N.A. soil DNA kit (Omega Biotek, GA 30,071, USA). The extracted DNA was purified and quantified by spectrophotometer. (Thermo, MA 02,451, USA). The forward and reverse primers were connected with the universal primer of Illumina Miseq high-throughput sequencing platform, and the PCR products with universal primer sequence at both ends were obtained by the first round PCR reaction using genomic DNA as template, and the PCR products obtained in the first round were purified. The PCR product of the sequence was obtained by connecting the two ends of the sequencing tag sequence with the primer sequence matched with the general primer sequence of the first round of PCR. The purified PCR product was used as the template for the second round of PCR reaction. The amplicon extracted from 2% agarose gel was purified by PCR purification kit (Beckman, Indiana 46,268, USA) and quantified using a Qubit® 2.0 fluorimeter (Invitrogen, CA 92,008, USA). The Illumina MiSeq high-throughput sequencing platform was used for sequencing (Shanghai Sangon Biotechnology Co., LTD., Shanghai, China).

### SOC and SIC aanalysis

Shimadzu TOC-V (Japan) was used to determine the SOC and SIC. The TOC (total organic carbon) instrument uses combustion oxidation-non-dispersive infrared absorption method for total organic carbon. Using high purity air (N_2_ + O_2_) as carrier gas, quantitative soil samples were added to the total carbon (TC) and IC (inorganic carbon) reaction chamber, respectively. The TC and IC were measured under their respective working conditions, and the TOC was calculated (TOC = TC—IC). Test conditions: carrier gas (high purity oxygen) pressure: 300 kPa; flow rate: 500 ml/min. TC condition: temperature 900 °C, cobalt oxide platinum catalyst. IC condition: temperature 200 °C, 25% phosphoric acid (superior purity) is reactive acid. Glucose (superior purity) and anhydrous sodium carbonate (Reference Reagent) are used as standard samples of TC and IC respectively.

### Statistical analysis

The remote sensing data of China's land use are based on Landsat 8 remote sensing images and generated through manual visual interpretation. The interpretation data are available for free download on the website of Institute of Geographic Sciences and Natural Resources Research, CAS, Chinese Academy of Sciences and the data center (https://www.resdc.cn/data.aspx?DATAID=335) and created by ArcGIS 10.2 platform (http://www.esri.com/sofware/arcgis/arcgis-for-desktop) for the study area. The experimental data were expressed as mean ± standard deviation (SD). SPSS (SPSS Inc., Chicago, IL, USA; Norusis, 2008) was used for one-way analysis of variance (ANOVA), and the significant differences of SOC, SIC and enzyme activities were obtained. Duncan’s test was used to evaluate significance when *P* < 0.05. The graph was drawn by using the software package origin 8.5. Redundancy analysis (RDA) was carried out by Canoco5 software (Microcomputer Power, Inc., Ithaca, NY, USA).

## Results

### Soil physico-chemical properties

The soil physico-chemical properties of the six farmlands were summarized in Table 2. Mean values of rhizosphere soil pH in maize fields were lowest at M1 (8.52), medium at M2 (9.08), and highest at M3 (9.45). In rice fields, the mean pH values were lowest at R1 (8.57), medium at R2 (9.11) and highest at R3 (9.47). These trends (in both maize and rice fields) were positively related with ESP in each field, M1 had the lowest ESP value of 8.14%, and M3 had the highest value of 23.53%. The SSC content in maize and rice fields ranged from 0.24 to 0.52% and 0.23 to 0.45% respectively. The maximum occurs in M3 and R3. The content of SOM and AN decreased with the increase of soil pH and ESP.

**Table 2:** Physico-chemical properties of rhizosphere soil (ESP, exchangeable sodium percentage; SSC, soil salt content; SOM, soil organic matter; AN, Alkali-N).

Sampling sites	pH	ESP (%)	SSC (%)	SOM (g kg^−1^)	AN (g kg^−1^)
M1	8.52 ± 0.29	8.14 ± 0.22	0.24 ± 0.04	20.14 ± 0.15	0.11 ± 0.04
M2	9.08 ± 0.23	16.76 ± 0.18	0.37 ± 0.03	15.89 ± 0.26	0.10 ± 0.02
M3	9.45 ± 0.21	23.53 ± 0.21	0.52 ± 0.05	11.53 ± 0.25	0.08 ± 0.02
R1	8.57 ± 0.19	8.06 ± 0.25	0.23 ± 0.05	21.23 ± 0.23	0.12 ± 0.05
R2	9.11 ± 0.17	17.24 ± 0.17	0.38 ± 0.04	14.52 ± 0.22	0.11 ± 0.03
R3	9.47 ± 0.23	22.65 ± 0.19	0.45 ± 0.03	11.19 ± 0.26	0.09 ± 0.03

### Soil carbon distribution

**Figure 2:**
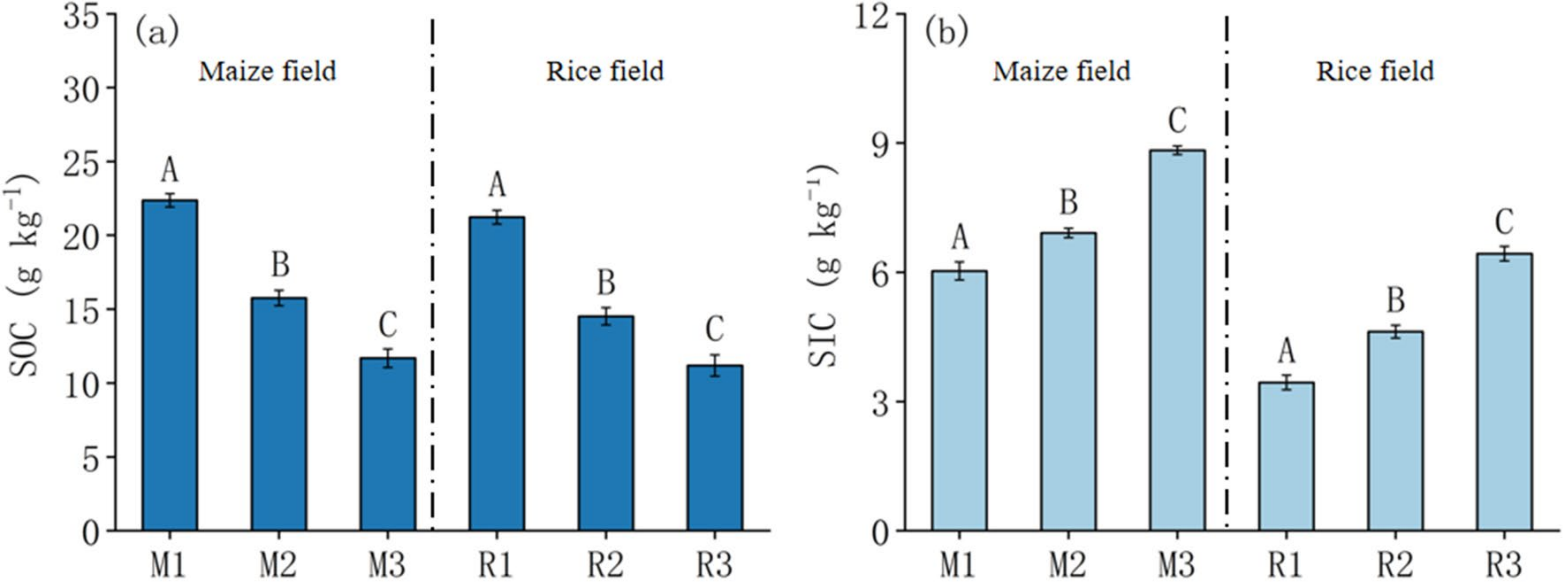
SOC and SIC distribution in the rhizosphere soil of maize (M) and rice (R) in different saline–alkali fields. Capital letters indicate the significance of the same crop in different fields(*P* < 0.05).

SOC in maize fields and paddy fields decreased with the increase of soil salinity (Fig. 2a). SOC in maize field was 11.68–22.37 g kg^−1^, and that in paddy field was 11.19–21.23 g kg^−1^. SIC in maize field and rice field increased with the increase of soil salinity. SIC in maize and rice were 6.03–8.83 g kg^−1^ and 3.44–6.43 g kg^−1^, respectively (Fig. 2b). SOC was significantly different in maize fields and rice fields, the same as SIC (*P* < 0.05).

### Soil enzyme activities

The soil enzyme activities of maize and rice decreased with the increase of salinity (Table 3). The activities of catalase, invertase, amylase and *β*-glucosidase in maize fields were 4.38–5.60 mg g^−1^, 4.54–6.14 mg g^−1^, 0.96–1.39 mg g^−1^, 0.52–0.57 mg g^−1^, respectively. The results of soil enzyme in paddy field were 6.29–7.58 mg g^−1^, 3.41–5.62 mg g^−1^, 1.45–1.89 mg g^−1^, 0.44–0.49 mg g^−1^, respectively. The amylase and catalase activities in rice fields were higher than those in maize fields. ANOVA revealed that there were significant differences in soil enzyme activities between maize fields with different salinity levels, and there were significant differences between different enzyme activities in the same sampling site. The analysis results of rice and maize samples were consistent.

**Table 3:** Soil enzyme activities in the rhizosphere soil of maize (M) and rice (R) in different saline–alkali fields.

Sampling sites	Catalase (mg g^−1^)	Invertase (mg g^−1^)	Amylase (mg g^−1^)	*β*-glucosidase (mg g^−1^)
M1	5.60 ± 0.14Aa	6.14 ± 0.08Ab	1.39 ± 0.02Ac	0.57 ± 0.03Ad
M2	4.97 ± 0.21Ba	5.54 ± 0.06Bb	0.99 ± 0.02Bc	0.54 ± 0.01Ad
M3	4.38 ± 0.11Ca	4.54 ± 0.06Cb	0.96 ± 0.03Bc	0.52 ± 0.04Ad
*P*1	7.58 ± 0.09Aa	5.62 ± 0.05Ab	1.89 ± 0.05Ac	0.49 ± 0.03Ad
*P*2	6.54 ± 0.11Ba	5.30 ± 0.08Bb	1.81 ± 0.04Ac	0.49 ± 0.03Ad
*P*3	6.29 ± 0.16Ba	3.41 ± 0.03Cb	1.45 ± 0.04Cc	0.44 ± 0.01Ad

Capital letters indicate the significance of the same crop in different fields(*P* < 0.05) and lowercase letters indicate the difference in soil enzyme activities of the same crop in the same field (*P* < 0.05).

### Diversity index of soil microbial community

The Shannon index and Simpson index of soil microorganisms can accurately reflect the characteristic function of microbial community diversity of this component. There were differences in the diversity index of soil microbial community structure between saline–alkali maize and rice fields. Coverage index actually reflects whether sequencing results represent the real situation of the samples. The coverage of all samples was above 93%, which indicated that the results of sequencing were relatively reliable and basically reflected the situation of soil bacteria. The Shannon, ACE and Chao1 indexes of the six sampling sites revealed a similar trend, which were M1 > M2 > M3, *P*1 > *P*2 > *P*3 (Table 4).

**Table 4:** Characteristics of soil bacterial richness and diversity indexes in the rhizosphere soil of maize (M) and rice (R) in different saline–alkali fields.

Sampling Sites	Shannon index	ACE index	Chao1 index	Coverage	Simpson index
M1	7.54	34,852	23,036	0.94	0.01
M2	7.31	17,410	12,868	0.95	0.03
M3	7.02	15,948	10,885	0.93	0.05
P1	8.00	32,016	21,388	0.94	0.01
P2	7.70	29,489	20,566	0.93	0.02
P3	7.08	19,368	11,617	0.95	0.05

### Bacterial community structure

**Figure 3:**
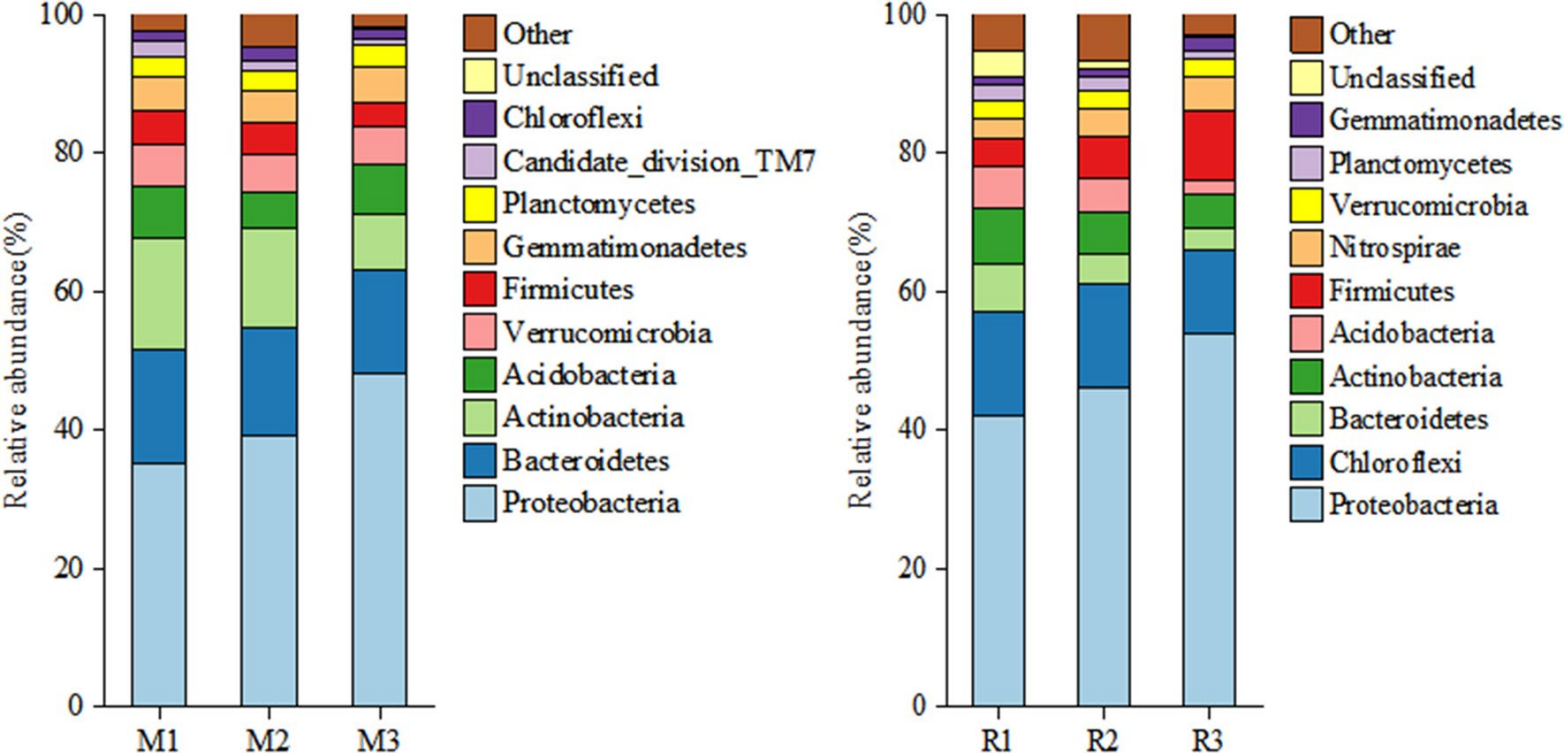
At the phylum level, soil bacterial community composition in the rhizosphere soil of maize (M) and rice (R) in different saline–alkali fields.

The bacterial 16S rRNA genes collected from sampling sites were sequenced by high-throughput sequencing and cluster analysis. According to the relative abundance of bacteria at the phylum level (Fig. 3), saline–alkali stress significantly affected the rhizosphere soil bacterial community composition. At the phylum level, the main groups of soil bacterial communities in saline–alkali maize fields were *Proteobacteria*, *Bacteroidetes*, *Actinobacteria, Verrucomicrobia*, and the relative average abundance ratios were 40.08%, 15.68%, 12.80%, 6.69%, and 5.50%, respectively (Fig. 3). In the rhizosphere soil of maize (M), the sum of the relative abundance of the five bacterial phyla accounted for 81.54% of the total number of soil bacteria. *Proteobacteria* and *Chloroflexi* were the main groups of bacterial communities in saline–alkali rice fields at the phylum level, accounting for 61.33% of the total bacterial sequences recovered (47.33 and 14.00%, respectively) (Fig. 3). The other major phyla (average relative abundance > 1%) were *Firmicutes* (6.67%), *Actinobacteria* (6.33%), *Bacteroidetes* (4.80%), *Acidobacteria* (4.33%), *Nitrospirae* (4.00%), *Verrucomicrobia* (2.63%) and *Planctomycetes* (1.87%). The sum of relative abundance of five phyla accounted for 91.96% of the total number of soil bacteria in the rhizosphere soil of rice (R). The relative average abundance ratio of *Bacteroidetes* and *Actinobacteria* in maize rhizosphere soil was 10.8% and 6.4% higher than that in rice rhizosphere soil, respectively, while the relative average abundance ratio of *Chloroflexi* in rice rhizosphere soil was 12.3% higher than that in maize rhizosphere soil.

**Figure 4:**
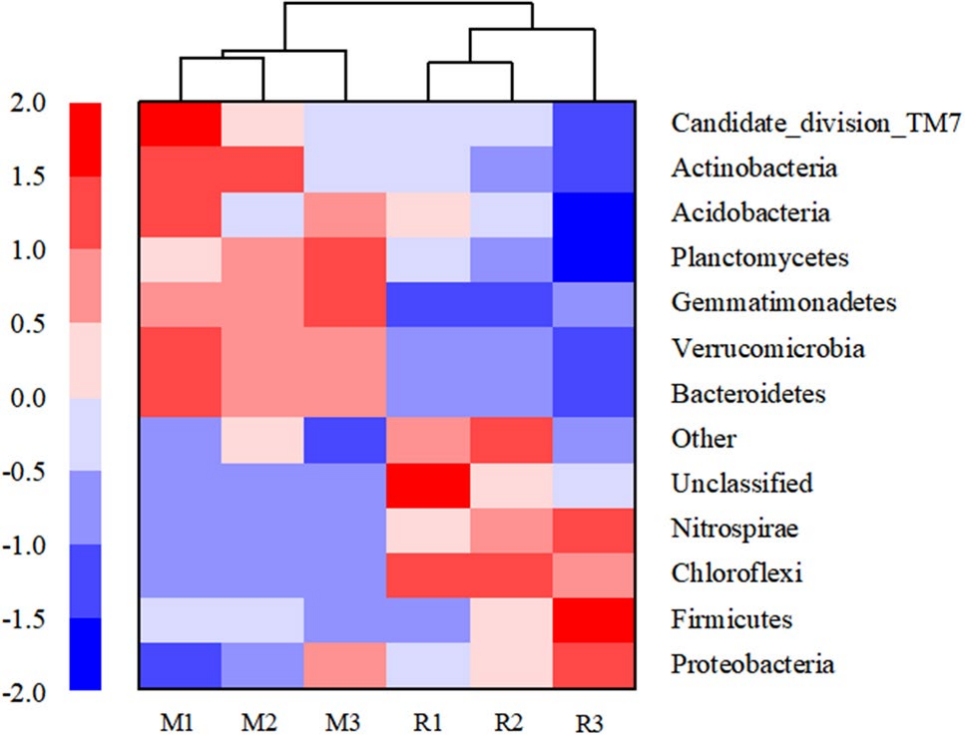
Heat map of microbial community structure in the rhizosphere soil of maize (M) and rice (R) in different saline–alkali fields.

The heat map of microbial communities at the phylum level showed that the abundance of bacteria in rhizosphere soil samples of maize and rice was different in different saline–alkali fields. The results of cluster analysis showed that the discrepancy between the relative and absolute abundances of bacteria in maize and rice rhizosphere samples in different saline–alkali fields (Fig. 4). Based on the results of cluster analysis the samples of M1 and M2, R1 and R2 were clustered into two close groups, respectively, while the samples of M3 and R3 were relatively far away from those groups (Fig. 4).

## Discussion

### Effects of physico-chemical properties on the variations of SOC and SIC

**Figure 5:**
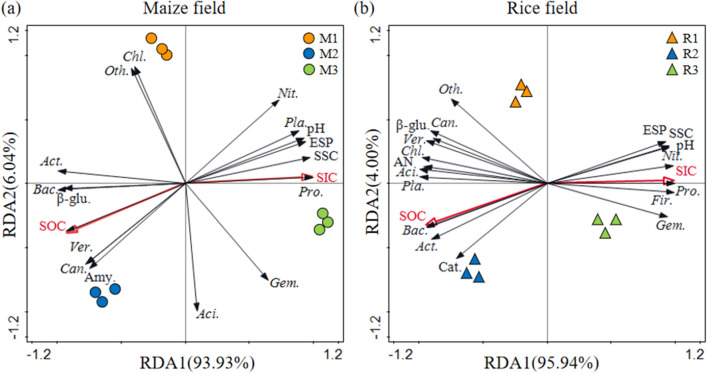
Redundancy discrimination analysis (RDA) depicting the relationship between the main soil physico-chemical parameters, soil enzymes and microorganism in the rhizosphere soil of maize (M) and rice (R) in different saline–alkali fields (Nit., Nitrospirae; Pla., Planctomycetes; Pro., Proteobacteria; Gem., Gemmatimonadetes; Aci., Acidobacteria; Chl., Chloroflexi; Oth., Other; Act., Actinobacteria; Unc.,Unclassified; Fir., Firmicutes; *β*-glu., *β*-glucosidase; Can., Candidate-division-TM7; Ver., Verrucomicrobia; Bac., Bacteroidetes; Amy., Amylase; Cat., Catalase).

Soil physico-chemical properties are affected not only by SOC, but also by soil mineral composition, especially SIC. Soil pH is the most sensitive indicator to regulate the cycle and sequestration of SOC, and to affect crop growth, soil microbial and enzyme activities^29–31^. The optimal pH of bacteria was 6.5–8, and that of fungi was 5–6^32^. The soil pH of M3 and R3, is 9.36 and 9.32, respectively, which inhibits soil microbial activity, thus affecting the humification process of roots and litter, and hindering the input and accumulation of SOC^33^. The reason for the high pH is that the sodium ions adsorbed on the soil colloid hydrolyze and produce OH^−^ ions, which increase the alkalinity of the soil. In the case of saturated soil colloids, the exchange of sodium hydrolysis may cause soil alkalinity reaction. There was a significant correlation between ESP and pH, and a negative correlation between ESP and SOC (Fig. 5) indicating that higher ESP was not conducive to SOC sequestration. In the process of soil root development, SOM will be secreted to increase SOC content. Because there are more carbonate and bicarbonate in the soil, the higher soil pH also promotes the accumulation of SIC. SOC had a negative correlation with SIC, that is, the content of SIC in rhizosphere soil decreased with the increase of SOC content, which was consistent with the research results of Huang et al.^34^.

### Effects of enzyme activities on the variations of SOC and SIC

Determination of enzyme activities, including catalase, invertase, amylase and *β*-glucosidase, in the rhizosphere soil of maize and rice in different saline–alkali fields is helpful to understand soil biochemical reactions^35^. The results showed that the activities of catalase, invertase, amylase and *β*-glucosidase decreased with the increase of salinity (Table 3). Saline–alkali stress changes the structure and function of cell membrane and has toxic effects on cells. At the same time, it increases the soil osmotic pressure, hinders the absorptive capacity of plants, limits the growth of plants, makes the root system cannot fully develop, directly affects the secretion of enzymes, leading to great differences in enzyme activities in different soil regions^36^. The increase of SSC will reduce the activities of soil enzymes. Compared with CaCl_2_ and Na_2_SO_4_, NaCl has a stronger inhibitory effect on soil enzymes and microbial activities^37^. However, under high saline–alkali stress conditions, enzyme can still promote the conversion of soil carbon. The results showed that the activity of amylase and catalase in rice field was higher than that in maize field, which may be due to the fact that rice was in the irrigation stage, and the hydrothermal conditions promoted the growth and reproduction of microorganisms. Under suitable growth conditions, plant roots will secrete more organic acids and carbohydrates, stimulating the activities of catalase and amylase. In the process of catalyzing the decomposition of hydrogen peroxide, soil catalase will release oxygen, which is conducive to the life activities of aerobic microorganisms in the soil, thus increasing the soil humification intensity and SOC content. Amylase catalyzes SOM to increase SOC content in soil^38^. Compared with maize, rice rhizosphere soil is in the state of water for a long time, which is easy to lead to a large amount of hydrogen peroxide accumulation, so the soil catalase is more active.

### Effects of microorganisms on the variations of SOC and SIC

In the mature stage of maize and rice, the water and heat conditions are suitable, the rhizosphere exudates provide nutrients for microorganisms, and the plant growth and soil quality tend to be stable. Bacteria changed the most under different saline–alkali degree and were always the dominant microorganisms in saline–alkali soil. It was found that at the phylum level, a variety of bacteria decreased with the increase of salinity. It may be that *Firmicutes*, *Proteobacteria* and *Nitrospirae* will increase sharply in adversity^39,40^. And they were significantly affected by pH and ESP, and were positively correlated with SIC in saline–alkali rice rhizosphere soils (*P* < 0.05) (Fig. 5). The main reasons for community diversity difference were that the higher salinity inhibited the number and activity of microorganisms and the inhomogeneity of microbial species. With the increase of salinization degree, the activity and population number of microorganisms decreased under the adverse growth conditions of saline–alkali soil, which reduced the amount of plant derived SOC fixed into soil by microorganisms^41–43^. In the long run, the carbon release rate of slow circulation pool has an important impact on ecosystem carbon storage during salinization^44^. Microorganisms play a key role in the formation of carbonate. Microorganisms can accelerate karstification and promote CO_2_ deposition. It can also produce acid through metabolic activities, resulting in dissolution of carbonate and release of CO_2_. Microorganisms can also form carbonate from CO_2_ produced by respiration. Microbial community plays a key role in the decomposition and transformation of soil carbon. *Proteobacteria*, *Actinobacteria*, *Bacteroidetes* and *Acidobacteria* are relatively high in maize and rice rhizosphere soils, which may contribute to nutrient uptake by root system and maintain the balance of microenvironment, so as to improve the soil environment and enhance the ability to resist saline–alkali stress. Therefore, they are valuable resources for biological improvement of saline–alkali soil regions.

## Conclusions

1. Saline–alkali stress changed rhizosphere soil physico-chemical properties and affected soil enzyme activities. The activities of catalase, invertase, amylase and *β*-glucosidase decreased with the increase of salinity, i.e., M1 > M2 > M3, R1 > R2 > R3, with significant differences (*P* > 0.05), and the activities of amylase and catalase in rice rhizosphere soil was higher than that in maize rhizosphere soil.
2. Saline–alkali stress changes the community structure of rhizosphere soil. At the phylum level, most of bacteria decreased with the increase of salinity. On the contrary, compared with M1, the relative abundance of *Proteobacteria* and *Firmicutes* in M2 and M3 increased by 16.86% and 48.43%, 34.50% and 46.19%, respectively. Compared with R1, the relative abundance of *Firmicutes*, *Proteobacteria* and *Nitrospirae* in R2 and R3 increased by 47.50% and 142.50%, 9.52% and 28.57% and 36.67% and 76.67%, respectively.
3. There was a significant correlation between ESP and pH, and a negative correlation with SOC (*P* < 0.05) of maize and rice rhizosphere soil in soda saline–alkali field. *Proteobacteria*, *Gemmatimonadetes*, *Planctomycetes* and *Nitrospirae* were significantly affected by pH and ESP, and were positively correlated with SIC in saline–alkali maize and rice rhizosphere soils (*P* < 0.05).

## Data Availability

All data generated or analysed during this study are included in this published article.
